# Single cell organization and cell cycle characterization of DNA stained multicellular tumor spheroids

**DOI:** 10.1038/s41598-021-96288-6

**Published:** 2021-08-23

**Authors:** Karl Olofsson, Valentina Carannante, Madoka Takai, Björn Önfelt, Martin Wiklund

**Affiliations:** 1 Department of Applied Physics, Science for Life Laboratory, KTH Royal Institute of Technology, Stockholm, Sweden; 2 Department of Microbiology, Tumor and Cell Biology, Karolinska Institutet, Solna, Sweden; 3 Department of Bioengineering, University of Tokyo, Tokyo, Japan

**Keywords:** Cancer imaging, Cancer microenvironment, Cancer models, Cellular imaging, Nuclear organization, Image processing

## Abstract

Multicellular tumor spheroids (MCTSs) can serve as in vitro models for solid tumors and have become widely used in basic cancer research and drug screening applications. The major challenges when studying MCTSs by optical microscopy are imaging and analysis due to light scattering within the 3-dimensional structure. Herein, we used an ultrasound-based MCTS culture platform, where A498 renal carcinoma MCTSs were cultured, DAPI stained, optically cleared and imaged, to connect nuclear segmentation to biological information at the single cell level. We show that DNA-content analysis can be used to classify the cell cycle state as a function of position within the MCTSs. We also used nuclear volumetric characterization to show that cells were more densely organized and perpendicularly aligned to the MCTS radius in MCTSs cultured for 96 h compared to 24 h. The method presented herein can in principle be used with any stochiometric DNA staining protocol and nuclear segmentation strategy. Since it is based on a single counter stain a large part of the fluorescence spectrum is free for other probes, allowing measurements that correlate cell cycle state and nuclear organization with e.g., protein expression or drug distribution within MCTSs.

## Introduction

Solid cancer tumors are complex 3-dimensional (3D) environments where multiple cell types embedded in extra-cellular matrix interact through direct cell–cell contact or soluble factors^1,2^. Biochemical and biophysical factors are ingredients that together constitute the tumor microenvironment which influence cell behavior and cell state^3^. Standard in vitro 2D cell culture techniques based on seeding cells on flat plastic surfaces do not capture several important aspects of a solid tumor^4^.

A popular approach to introduce a higher complexity into in vitro cell experiments is to use 3D cell cultures where the additional dimension reduces the disparity to in vivo conditions. One type of 3D cancer cell culture is multicellular tumor spheroids (MCTSs) which are spherical aggregates of one or several cell types. They are appreciated for combining the advantages of regular 2D culture (robustness and reproducibility) while introducing important 3D physiological aspects of solid tumors such as gas- and nutrient gradients^5^. These gradients are also found in many solid tumors due to poor vascularization and can influence cell state, drug delivery and therapy efficacy^6^. The inhomogeneous distribution of nutrients, gas and cellular waste, caused by the limited molecular diffusion between the MCTS surface and center, introduces a spatial dependency in cell behavior and state^7^. For example, the cell cycle depends on the position within the MCTS^8^. Flow cytometry-based studies using DNA content analysis is a traditional tool for cell cycle analysis but lacks spatial information^9^. Employing immunofluorescence or FUCCI transfected cells^10^, it has been shown that the cell cycle state depends on the distance from the MCTS surface which is important when investigating novel cancer drug treatments targeting cell cycle checkpoints^11^.

A major challenge when working with MCTSs is characterization and analysis. Flow cytometry can generate quantitative single cell data but requires high cell numbers and MCTS disaggregation which may introduce artefacts and eliminates the possibilities to capture spatial information^12^. Spatial information is also lost when working with single cell sequencing^13^, western blot^14^ and qRT-PCR^15^. To resolve cell position in combination with biologically relevant information, different microscopy-based imaging modalities, such as confocal, two-photon and light sheet microscopy^16^, are convenient characterization tools^17^. While single cell resolution imaging of whole MCTSs is achievable with microscopy, there are a few caveats that have to be addressed; light scattering in thick specimens, quantitative data retrieval and a limited fluorescence spectrum. Light scattering can be reduced by MCTS embedding and sectioning^18^ or mounting whole MCTSs in a refractive index matching solution (RIMS) which immerses the spheroid structure and removes all light scattering interfaces making the specimen transparent^19–22^. Image quality and resolution in light sheet microscopy can be further improved by a MCTS expansion protocol in combination with RIMS mounting^23^.

An attractive approach for quantitative single cell resolution data extraction is image analysis of fluorescently stained nuclei captured in microscopy image stacks. Fluorescently stained nuclei can be segmented and provide information about number of cells, position and nuclear volumetric parameters within the MCTS. While nuclear segmentation is an appealing target for image analysis due to ease-of-labeling and simple geometrical structure, the segmentation methods require the ability to resolve and handle tightly packed nuclei due to the high cell density in MCTSs. There are several commercial and freely available 3D nuclear segmentation software tools that are either based on machine learning or more classical methods^24^. Machine learning based nuclear segmentation is a quite fast and accurate method but require training sets which are not available in large quantities^25,26^. The more classical methods based on filter pre-processing steps and nuclear separation algorithms, such as watershed, are quite reliant on parametrization but can be tailored to produce good results for a certain set of data^27–29^. Other notable approaches include line-of-sight decomposition^30^ and gradient flow tracking^31^.

Even with a rich flora of available imaging, clearing and nuclear segmentation methods for MCTS analysis, there is still a need to fully exploit the fluorescence intensity, position and volumetric information of segmented nuclei and connect this information to cell state and biological function. An example of combining nuclear segmentation and light sheet microscopy was used in an investigation of cell division axis orientation and dynamics in live HTS116 MCTSs, which shows parts of the wide extent of information that can be extracted from analysis of a single fluorophore^32^.

In this study we outline a workflow using on-chip MCTSs formation and confocal imaging together with 3D image analysis in order to connect nuclear segmentation data to biologically relevant information using a single counterstaining fluorophore. By using a watershed-based nuclear segmentation script, we determined the cell cycle state of individual cells in a 3D cell culture through quantifying volumetric data and DNA content in DAPI-stained MCTSs made of the renal carcinoma cell line A498. DAPI was chosen in this study due to its widespread use as a counter stain and stochiometric binding to DNA^33^. DNA-content analysis is a standard tool for DNA ploidy characterization and has been shown to work with nuclear segmentation in thin tissue sections and 2D culture^34–36^ but, to our best knowledge, not in image stacks of whole cleared MCSTs. Compared to transfection or immunofluorescence, the single fluorophore staining used herein is a less labor-intensive cell cycle analysis approach with the additional benefit of leaving the rest of the fluorescence spectrum free for other staining targets within the MCTSs. We combine the image analysis methodology with a ultrasonic standing wave (USW)-based MCTS culture platform where MCTSs can be formed, cultured, stained, cleared and imaged on-chip^37^. Using this platform, the MCTSs are actively formed by the USW-based method and processed in a microwell chip optimized for high-resolution imaging in 3D, resulting in 100 homogeneously sized MCTSs cultured and analyzed in parallel.

To demonstrate the connection between the integrated DAPI intensity and cell cycle stage, we used FUCCI transfected and DAPI stained A498 MCTSs and found good agreement when comparing the expression levels of the cell cycle regulators cdt1 and geminin to the DNA content-dependent DAPI fluorescence intensity. We then used the volumetric and fluorescence data from the nuclear segmentation in combination with the FUCCI defined cell cycle stage to train a machine learning-based support vector machine (SVM) classifier to find S and G2 positive nuclei in the MCTSs. As a proof-of-principle, we quantify the spatial dependence of individual S/G2 positive cells in DAPI stained A498 MCTSs cultured for 24 and 96 h. We also illustrate how the distance between the MCTS perimeter and each individual nucleus can be used as a continuous variable, in combination with nuclear volumetric information, to investigate the inhomogeneous MCTS structural composition.

The methods outlined in this study demonstrate the possibilities for biological and structural single cell resolution data extraction in MCTSs using a simple clearing and counter-staining protocol, leaving the rest of the fluorescence spectrum free for other fluorescent probes. This methodology can in principle be used regardless of 3D nuclear segmentation and stochiometric DNA staining strategy and we believe it will be useful in MCTS drug screening applications and basic tumor biology research.

## Methods

### Cell lines and culture

The cells used in this study was renal carcinoma A498 cell line (ATCC© HTB-44™, ATCC) which was cultured in RPMI-1640 GlutaMAX (Thermo-Fischer Scientific) supplemented with 10% heat inactivated fetal bovine serum (Thermo-Fischer Scientific), 1 × MEM Non-essential amino acid solution and 25 mM HEPES (Thermo-Fischer Scientific). Cells were kept in T75 flasks and maintained at 37 °C in 5% CO_2_. Cells were regularly passaged before reaching confluency.

### Ultrasonic MCTS device and MCTSs formation

**Figure 1:**
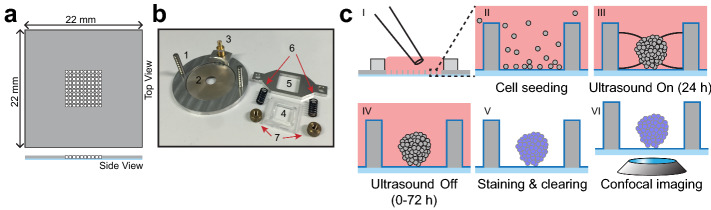
On-chip formation, culture, staining and imaging of MCTSs. The microwell plate (**a**) consists of a 22 × 22 × 0.3 mm^3^ silicon wafer with 100 etched microwells (350 × 350 µm^2^) bonded to a 170 µm thick coverglass. Ultrasonic radiation forces, used in microwell cell aggregation, were induced in a device (**b**) where the chip was placed on a transducer (1) with a piezo ceramic plate (2) connected to a SMB connector (3). The microwell chip was secured against the piezo by plastic (4) and aluminum (5) frames mounted with springs (6) and nuts (7). The workflow for MCTS culture and processing (**c**) starts with seeding cells (I) which sedimented into the microwells (II). Transducer actuation for 24 h induces ultrasonic radiation forces acting on the cells in each microwell causing aggregation (III). After 24 h of active ultrasound culture, the chip is further kept in passive culture for 0–72 h (IV) before on-chip staining, clearing (V) and imaging (VI).

The ultrasonic standing wave (USW) MCTS culture platform consist of a silicon-glass multiwell chip and a transducer fitted with chip clamping equipment which has previously been described in detail^37–39^. In short, the multiwell chip (Fig. 1a) was produced by dry-etching 100 square holes (350 × 350 µm^2^) with concave walls through a 300 µm thick silicon wafer before being bonded to a 170 µm glass wafer and diced into a 22 × 22 mm^2^ chip^40^. A PDMS gasket bonded around the microwell array provided a shared medium reservoir above the wells and liquid manipulation was performed with a regular pipette. Cell attachment to the glass bottom and silicon walls was prevented by a poly(2-methacryloyloxyethyl phosphorylcholine-*co*-3-methacryloxypropyl trimethoxysilane-*co*-3-(methacryloyloxy)propyl-tris(trimethylsilyloxy) silane) protein repellent polymer-coating which was stabilized at the surfaces through a combination of covalent bonding and hydrophobic interaction within the coating layer^41,42^. The crucial design feature of the chip is the glass bottom which thickness corresponding to a No. 1.5 coverslip giving excellent imaging properties.

The ultrasonic transducer (Fig. 1b) comprises of an aluminum frame fitted with a circular piezo ceramic plate coupled with soldered wires to a SMB connector. The multiwell chip was placed on the piezo ceramic plate with immersion oil as coupling medium and retained in position with plastic (Fig. 1b 4) and aluminum frames fixed with springs and nuts.

To form MCTSs (Fig. 1c), a single cell suspension (100 µL, 500,000 cells/mL) was seeded with a pipette into the medium reservoir and the cells were allowed to sediment into the microwells. The microwell chip was placed in the transducer platform which was actuated with a frequency modulation scheme (2.47 ± 0.05 MHz, 1 kHz sweep rate) which corresponds to a half-wavelength resonance condition in the microwells. The USW exerts acoustic radiation forces on the cells and trap them into aggregates in the pressure nodes located in the center of each well. The aggregates were stabilized and formed into spheroids during 24 h with the USW turned on (active USW culture). After 24 h, the multiwell chip was disassembled from the transducer and kept without ultrasound exposure (passive culture) in a regular incubator until fixation and staining (0–72 h).

### MCTS staining, FUCCI transfection and clearing

When the MCTSs had been in culture 24–96 h they were washed 3 times in PBS before fixation in BD Cytofix/Cytoperm™ solution (BD Biosciences), containing 4% paraformaldehyde, for 10 min and protected from light. The fixed MCTSs were washed another 3 times in PBS before three 5-min washes with a wash buffer (2% bovine serum albumin (BSA) and 0.2% Triton X-100 in PBS) to prepare the MCTSs for staining. The MCTSs were incubated in a stain solution (0.1% BSA and 0.2% Triton X-100 in PBS) with 5 µg mL^−1^ DAPI (Invitrogen) for 4 h. The staining was followed by three 20-min washes using wash buffer before proceeding to the clearing step. All staining steps were performed in room temperature and on-chip with the MCTSs retained in the microwells (Fig. 1b V).

For cell cycle characterization, the Premo™ FUCCI Cell Cycle Sensor (Invitrogen) was used for transient transfections. A498 cells were transiently transfected according to the manufacturer’s instructions with some slight adjustments to accommodate to the 3D culture. Mixed FUCCI kit was added to the single cell suspension at a concentration of 30 particles per cell before seeding into the microwell chip and transducer mounting. After 24 h of active USW culture, the FUCCI transfected MCTSs were fixed and stained with DAPI according to the protocol above.

To overcome the light scattering issues when imaging the MCTSs, a clearing protocol based on a refractive index matching solution (RIMS) was employed to render the MCTSs optically clear. The ready stained MCTSs were mounted in RIMS which for this study was 755 mg mL^−1^ Iohexol (Omnipaque 355 mg mL^−1^ Iodine, GE Healthcare). To avoid the MCTSs escaping the microwells due to the comparably high RIMS density and limit MCTS size changes due to rapid internal liquid exchange, the RIMS was introduced in the steps (10%, 25%, 50%, 75% and 100% (RIMS/PBS vol/vol)).

### Confocal imaging

Laser scanning confocal imaging (Zeiss LSM 880) was performed with the microwell chip mounted in a custom holder using a 63 × oil-immersion objective (Plan-Apochromat 63 × /1.40 Oil DIC M27, Zeiss) and a pixel resolution of 512 × 512, with the pixel dimension 0.4393 × 0.4393 µm^2^, in combination with a pinhole size corresponding to 0.9 µm thick optical sections to allow interpolation along the z-axis and isotropic voxel size in the nuclear segmentation stage. Laser intensity and image acquisition parameters were adjusted to avoid overexposed pixels throughout the MCTS volume. Optical sections were acquired with 0.9 µm section steps and the z-stacks covered the full MCTS volume.

### Nuclear segmentation

**Figure 2:**
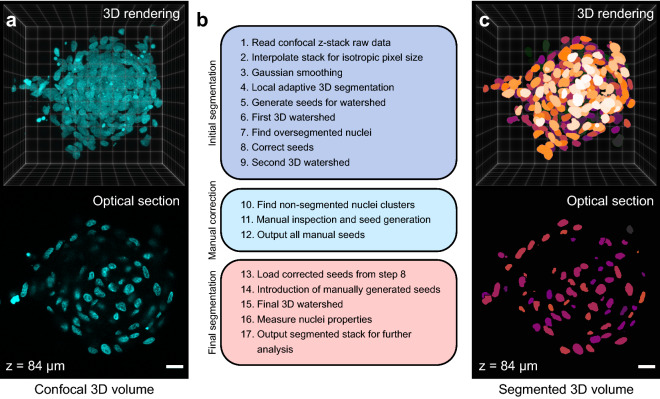
Nuclear segmentation of confocal z-stacks. The full volume of DAPI stained and Histodenz cleared A498 MCTSs was acquired by confocal microscopy (3D rendering and optical section at z = 84 µm) (**a**). Outline of an in-house developed MATLAB script (**b**) used to segment all individual nuclei (**c**) for further volumetric and fluorescence intensity analysis. Scalebars are 20 µm.

Confocal image z-stacks of DAPI stained and cleared MCTSs (Fig. 2a) were loaded into MATLAB-scripts (Supporting information 1) that segmented the nuclei in 3 stages; initial segmentation, manual correction of seeds and a final segmentation (Fig. 2b). All the operations were done in 3D and not sequentially on each individual optical section.

To prepare the raw data for initial segmentation, the image stack was interpolated in the z-direction to achieve isotropic voxel size and a gaussian filter was applied to smooth the image volume. A local adaptive threshold algorithm segmented the background and foreground based on local voxel mean intensity in a neighborhood volume corresponding to an eighth of the interpolated stack volume. A set of morphological operations and volume filtering on the thresholded volumes closed all the holes and removed small objects.

The watershed algorithm was used in steps to separate nuclei clusters based on seeds. The first set of seeds were generated by finding regional minima after applying a distance and a H-minima transformation to the thresholded MCTS volume. After imposing the seeds as catchment basins in the distance transformed image stack, the watershed algorithm separated objects based on the seeds. To limit over segmentation, a second watershed was applied after removing seeds generating objects smaller than 70% of the median object volume (Supplementary Fig. 1a).

Object clusters still not separated into individual nuclei was identified by having a volume 40% larger than the median object volume and manually inspected (Supplementary Fig. 1b). In a graphical user interface showing the individual clusters, manual seeds were generated by the operator clicking in the image. The manually produced seeds replaced the seeds in the large object clusters (Supplementary Fig. 1c) and a final watershed was applied (Fig. 2c). All the nuclear volumetric and intensity parameters were acquired and exported together with the original image and final segmented image stack for further analysis.

### Nuclear segmentation evaluation

The nuclear segmentation performance was evaluated manually by inspection. The manual seed correction step before the final watershed was ensuring that most of the nuclei clusters were separated correctly. Only nuclei with a size between 60 and 180% of the median nucleus size was used in the post-segmentation analysis.

## Results

### Cell cycle and DAPI fluorescence correlation

**Figure 3:**
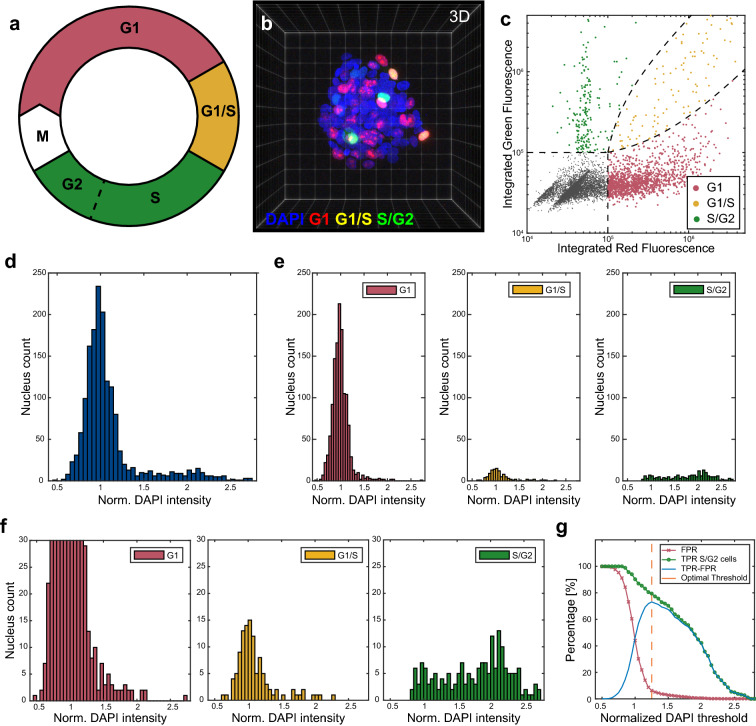
Correlation between cell cycle and integrated DAPI fluorescence in segmented nuclei. The cell cycle phases G1 (red), transition between G1/S (yellow) and G2/S (**a**) can be studied with the FUCCI construct which was transfected in A498 MCTSs (N = 40) (**b**). Based on integrated red and green intensity within each segmented nucleus, cells were classed as G1, G1/S or S/G2 positive (**c**). The FUCCI positive distribution of normalized integrated DAPI intensity per nucleus (**d**) could then be divided based on cell cycle position (**e**–**f**). Normalized DAPI intensity threshold against percentage of false positive rate (FPR) (red line), true positive S/G2 rate (TPR) (green line) and the difference between TPR and FPR (blue line) (**g**). The optimal threshold (1.25) is indicated at the TPR-FPR maximum (dashed orange line).

To characterize the cell cycle with singe cell resolution in MCTSs, we used transiently FUCCI transfected A498 MCTSs in combination with a DAPI counter-staining. Whereas a FUCCI transfected cell will go from expressing RFP tagged cdt1 in G1 to GFP tagged geminin in S and G2 (Fig. 3a), with a brief period of expressing both in the G1 to S transition, it is also possible to use DAPI, stoichiometrically bound to DNA, for DNA content-based cell cycle analysis. Cells in the G1 phase will have a single set of chromosomes (diploid, 2N) while a cell in G2 and M has double setup of DNA (4N) which gives twice the DAPI intensity. Cells in S will report a DNA content in between the 2N and 4N.

To investigate the possibility of using DNA-content analysis as a cell cycle predictor in MCTSs, DAPI-stained and transiently FUCCI transfected A498 MCTSs (n = 40) were cleared and imaged with confocal microscopy to capture the full MCTS volume (Fig. 3b). After nuclear segmentation with volume filtration to discard non-separated nuclei clusters, polyploid (> 4N) cells and small debris (Supplementary Fig. 2a), the RFP and GFP intensities were measured in each nucleus. Nuclei only expressing RFP was defined as G1 cells (n = 1291), GFP positive cells as S/G2 cells (n = 185) and cells with both RFP and GFP expression was identified as cells in the transition between G1 and S (n = 111) (Fig. 3c). Cells that had no GFP or RFP expression was defined as non-transfected (n = 4698) and the FUCCI transfection efficacy was calculated to 33.8%.

The integrated DAPI voxel intensity was measured in each individually segmented nucleus. Even though an efficient MCTS clearing was achieved, the integrated DAPI intensity was corrected linearly with respect to imaging depth (Supplementary Fig. 3). After linear correction, the integrated DAPI intensity was normalized to the median integrated intensity of all segmented nuclei in each MCTS before being pooled. The integrated DAPI intensity per nucleus histogram for all transfected cells (Fig. 3d) shows a clear peak around 1, indicating that most cells were diploid (one set of chromosomes, 2N). A smaller peak can be found around 2 which suggest that these cells contained double the amount of DNA (4N) while the normalized DAPI intensity for some cells were in between 1 and 2. Using the FUCCI positive cells, we investigated whether the DNA-content histograms were predicative for the cell cycle stage by studying the normalized DAPI intensity per nucleus histogram for G1 (red), G1/s (yellow) and S/G2 cells (green) which showed that the majority of diploid cells were G1 or G1/S cells while the S/G2 positive cells were distributed as 2N, 4N and ranging in between (Fig. 3e).

While the G1 and G1/S phase cells were distributed around 2N in the normalized DAPI intensity per nucleus histogram, the distribution tails towards the 4N cells (Fig. 3f). Therefore, determining a DAPI intensity threshold to classify the cell cycle stage of cells was not straight forward. To evaluate the feasibility of a DAPI intensity threshold as tool for classifying S/G2 cells, we plotted the true positive rate (TPR, green line) and false positive rate (FPR, red line) as a function of normalized DAPI intensity threshold (Fig. 3g). The optimal threshold (dashed orange line) was found to be 1.25 by identifying the maximum in the TPR subtracted by FPR curve (blue line). Using this threshold and comparing the classification result to the FUCCI classified ground truth it was found that the overall accuracy was 91.1%, TPR 79.8%, FPR 6.3% and the precision 62.6% for the S/G2 positive cells (Supplementary Fig. 4).

### Support vector machine cell cycle classification

**Figure 4:**
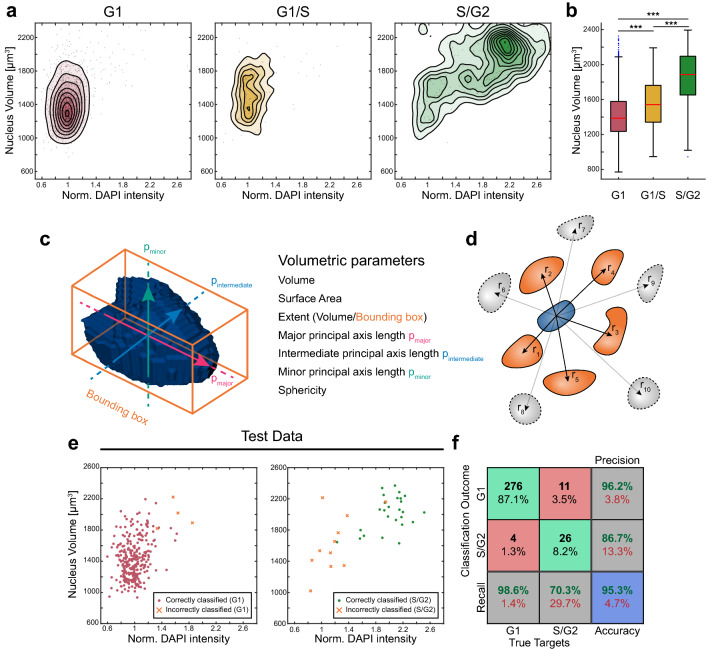
A machine learning-based support vector machine (SVM) trained on volumetric data and integrated DAPI intensity improves S/G2 classification. Density-scatter plots of normalized DAPI intensity against volume for each nucleus in G1 (red), G1/S (yellow) and S/G2 (green) (**a**). Volume boxplots for G1, G1/S and S/G2 showing 25th and 75th percentiles with median marked with red lines (**b**). Whiskers shows the furthest observation within 1.5 times the interquartile length away from the box edge and outliers are indicated by blue dots. Pairwise significance determined by Mann–Whitneys U-test and overall significance by Kruskal–Wallis (***p < 0.0001). A SVM was trained on the integrated DAPI intensity in combination with volumetric data (**c**) from FUCCI positive cells (N = 1270). Each volumetric parameter for a nucleus (blue) was normalized against the mean properties of the 5 closest nuclei (r_1-5_, orange) to limit spatial SMV dependencies (**d**). Scatterplots of integrated DAPI intensity per nucleus against volume shows correctly classified G1 (red) and S/G2 (green) cells as dots and misclassified cells as orange crosses for unseen test data (**e**). Confusion matrix describing the SVM classification performance shows the correctly (green) and incorrectly (red) classified cells, recall (true positive and false negative rate), precision (positive predictive value and false discovery rate) and overall accuracy (blue) for unseen test data (**f**).

While a simple normalized DAPI intensity threshold could to some degree classify cells in the S and G2 cell cycle phases, there were some nuclear volumetric parameters that also correlated with the cell cycle phase. One of the more striking examples was the nucleus volume where the S/G2 phase cells were larger compared to the G1 and G1/S cells (Fig. 4a,b). From the density-scatter plots it can also be appreciated that the 2N S/G2 phase cells (normalized integrated intensity ≈ 1) were very similar in volume to both the G1 and G1/S cells.

Using the built-in MATLAB function regionprops3 in the image processing toolbox, a multitude of volumetric parameters were acquired from analyzing the nuclear segmentation objects such as the nucleus volume, surface area and extent which is defined as the volume divided by the bounding box (the smallest cuboid enclosing the nucleus) (Fig. 4c). The principal axis lengths p_major_, p_intermediate_ and p_minor_ were the major axes of the ellipsoid with the same normalized second central moments as each segmented nucleus. We also calculated the sphericity of each nucleus using the volume and surface area where a perfect sphere would have the sphericity 1.

Taking advantage of both the volumetric parameters and the integrated DAPI intensity as predictor training data, a support vector machine (SVM) with a radial basis function kernel was trained in MATLAB with the FUCCI defined G1 and S/G2 phase cells as response training data. A support vector machine is a supervised learning model that creates a set of hyperplanes in a high-dimensional space which aims for maximum data separation in classification problems. The radial basis function kernel was preferred over a linear kernel since it is less sensitive to the collinearity in the predictor parameters found using Belsley collinearity test in MATLAB^43,44^. Soft margins were implemented during the SVM training. Since the cell position within MCTS might influence nuclear shape and volume, the volumetric parameters listed above were normalized to the five closest neighboring nuclei to avoid training the SVM on spatial information (Fig. 4d). Using 80% of the available FUCCI classified nuclei (n = 1270) a Bayesian hyperparameter optimization with a fivefold cross-validation of the SVM model was performed and benchmarked against three other algorithms implemented in MATLABs Statistical Toolbox; random forest, k-nearest neighbor and adaBoost with decision trees (Supplementary Fig. 5a,c,d). The benchmarking against other classification approaches showed that the SVM model performed equally and better compared to the other models in terms of balanced accuracy, overall accuracy, precision and recall. The balanced accuracy was calculated as TPR + TNR/2 to take the imbalanced data (G1 ≫ SG2 positive nuclei) into account. The robustness of the SVM model was also evaluated by dividing the FUCCI positive dataset based on multiwell microplates imaged on different days (n_microplates_ = 3). With the data divided by microplate (n_plate1_ = 222, n_plate2_ = 764 and n_plate3_ = 416), we optimized the hyperparameters, trained and tested using all possible data permutations (Supplementary Fig. 5b). The balanced and overall accuracy for training and testing was calculated from each permutation.

The same set of predictor and response data used in the SVM model evaluation was used in the final SVM training (Supplementary Fig. 4e,f), leaving the remaining 20% as unseen test data. The trained SVM model performance on the unseen test data was visualized by plotting the normalized DAPI intensity against nucleus volume for the correctly classified G1 (red dots) and S/G2 cells (green dots) and the misclassified cells (orange crosses) (Fig. 4e). Visual inspection showed that the SVM model was unable to classify S/G2 positive cells that were very similar to G1 cells in terms of volumetric and DAPI intensity. The test data confusion matrix of the true targets and classification outcome showed the correctly (green) and incorrectly (red) classified G1 and S/G2 cells (Fig. 4f). The determined overall accuracy was 95.3% (blue) while the S/G2 TPR was 70.3%, FPR 1.4% and the precision 86.7%.

### Spatial distribution of S/G2 positive cells in MCTSs

**Figure 5:**
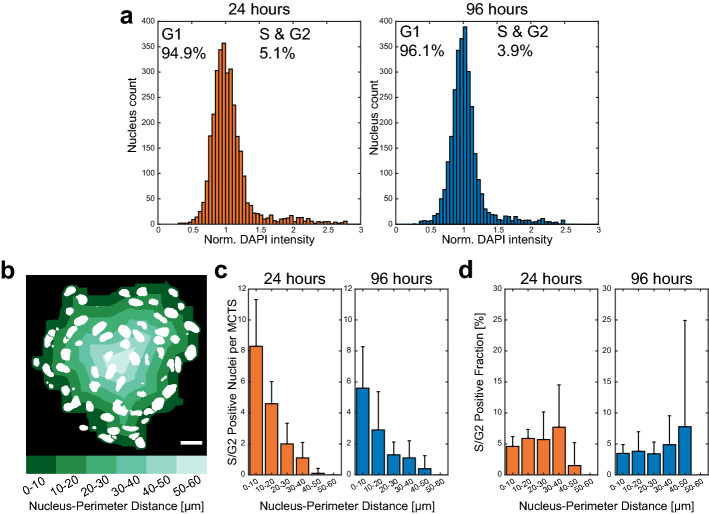
Spatial distribution of S/G2 positive cells in A498 MCTSs cultured for 24- and 96-h. Normalized integrated DAPI intensity per nucleus data of segmented nuclei from A498 MCTSs cultured for 24 h (N = 10, orange) and 96 h (N = 10, blue) (**a**). The integrated intensity and volumetric parameters were used to classify G1 and S/G2 cells (percentages in each histogram). The surface of each MCTS was approximated and the distance between each nucleus and the MCTS perimeter was measured and placed in concentric layers automatically using MATLAB (**b**). The number of S/G2 positive cells (**c**) and fraction of S/G2 positive cells (**d**) was calculated for each concentric 10 µm wide layer, from 0 to 60 µm, for each MCTS. Bar plot distributions show mean and standard deviation for 24 (orange) and 96 (blue) hours old MCTSs. Scalebar is 20 µm.

We used the nuclear segmentation script and SVM model to assess single cell volumetric and DAPI fluorescence intensity data for each nucleus to find cells in S/G2 phase (Fig. 5a). A498 MCTSs cultured for 24 h (n = 10) or 96 h (n = 10) were fixed, DAPI stained, cleared and imaged. The MCTS diameters approximately ranged from 120 to 150 µm and contained around 300 cells (Supplementary Fig. 6). The SVM model classified 5.1% and 3.9% of the cells in the MCTSs cultured for 24 h and 96 h respectively as being in S/G2 phase.

It is reasonable to assume that gas- and nutrient gradients, cell packing density and other parameters potentially affecting cell proliferation rate in MCTSs are distributed from the MCTS perimeter to the MCTS center. Therefore, we determined the distance relative to the MCTS surface for each nucleus. Cells that were detached from the MCTSs were filtered out and the MCTS surface was approximated by a morphological close operation on all remaining nuclei. Each MCTS was differentiated into 10 µm wide concentric layers based on distance to the perimeter and utilizing the nuclei centroids (segmented center of mass), each cell was placed in one of the layers (Fig. 5b). Treating all MCTSs individually, the number of S/G2 phase cells in each layer was counted and the largest number of cells was found in the outer layer (0–10 µm) with decreasing cell numbers as a function of distance away from the MCTS perimeter (Fig. 5c). Since the total cell number in each layer scales with volume, where the outer layer had the largest volume, we also investigated the fraction of S/G2 cells in all layers and found that it was quite evenly distributed (Fig. 5d). It should be noted the 40–50 µm and 50–60 µm layer range contained very few cells since the volumes close to the MCTS centers were small. The low cell numbers in the inner regions for MCTSs cultured for 96 h made the fraction calculation volatile which can be appreciated by the high standard deviation in the 40–50 µm layer range (Fig. 5d).

### Spatial distribution of G1 nucleus volumetric parameters

The volumetric data from the nuclear segmentation can also be used to investigate aspects of cellular organization within the MCTSs. Out of a plethora of possible volumetric measures available for MCTS characterization (Supplementary Fig. 7) we identified nucleus volume, nuclear aspect ratio and nucleus alignment as the most interesting. Those parameters were studied as function of distance from the MCTS perimeter for all G1 phase cells.

**Figure 6:**
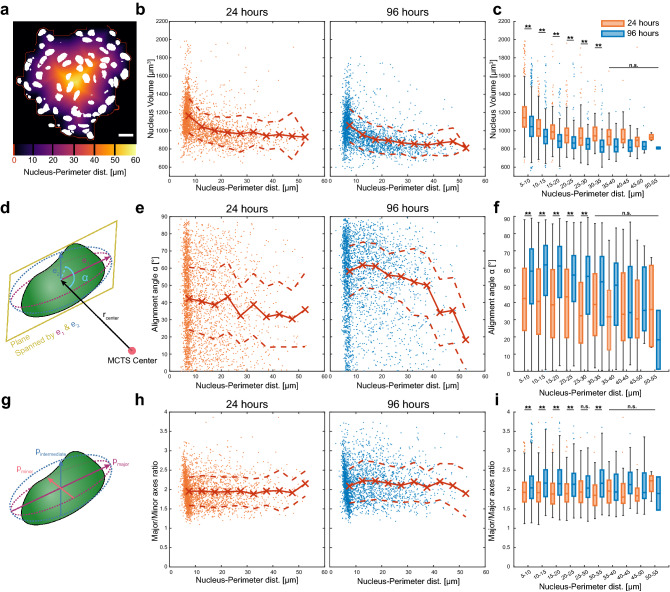
Nucleus volume and local cell density with nucleus-MCTS perimeter distance as a continuous variable. Scatter plots shows the nucleus-MCTS perimeter distance, as a continuous variable (**a**), against nucleus volume (**b**) for A498 MCTSs cultured for 24 h (orange dots) and 96 h (blue dots). The alignment angle α, calculated as the angle between the MCTS center-nucleus center vector (r_1_) and the plane spanned by the major (e_1_) and intermediate (e_2_) eigen vectors of the ellipsoid with the same second central momentum as the nucleus (**d**), is shown in the scatter plot against the nucleus-MCTS perimeter distance (**e**). The nuclear aspect ratio was studied by measuring the ratio between the major axis (p_major_) and minor axis (p_minor_) lengths (**g**) plotted against nucleus-MCTS perimeter distance (**h**). The nucleus-MCTS perimeter distance dependent trend in the scatter plots (red line with crosses) is calculated as the mean and median within 5 µm wide concentric layers for the nucleus volume, alignment angle and major/minor axes ratio respectively (**b**,**d**,**h**). Dotted line indicates the standard deviation and median absolute deviation. Box plot charts summarizes the data side by side for easier comparison (**c**,**f**,**i**). Significance was tested with two-way ANOVA followed by Tukey’s post-hoc multiple comparison (**p < 0.001). Scalebar is 20 µm.

Instead of placing cells in concentric layers as in the S/G2 phase quantification, we used the nucleus-MCTS perimeter distance as a continuous variable (Fig. 6a). From the same set of MCTSs cultured for 24 and 96 h as above, all nuclei classified as G1 were pooled based on time in culture. The volume against the nucleus-perimeter distance scatter plots shows a larger nucleus volume close to the MCTS surface compared to the inner parts (Fig. 6b). The red trend line in scatterplots was acquired by the mean volume in 5 µm thick radially concentric layers and dotted line shows the standard deviation. Overall, the nucleus volume and volume distribution were significantly lower after 96 h in culture compared to 24 h (Fig. 6c).

For each G1 nucleus, a 3D ellipsoid with a conserved second central momentum compared to the nucleus was used to acquire three eigenvectors corresponding to half the length and direction of the major, intermediate and minor axes of the ellipsoid. The eigenvectors can thus be used to study the nuclear orientation within the MCTS. We used the plane spanned by the major (e_1_) and intermediate (e_2_) eigenvectors and the vector r_center_ from the MCTS center to the nucleus center position to define the alignment angle as the angle between r_center_ and the eigenvector plane (Fig. 6d). The alignment angle is a measure of nucleus orientation in relation to the MCTS center where an alignment angle of 0° indicates nucleus aligned with the radius and 90° would be measured for a nucleus having its long axis oriented perpendicular to the MCTS radius (cf. p_major_ in Fig. 4c). This is further explained in Supplementary Fig. 8. The nucleus-perimeter distance against alignment angle scatter plots shows the distribution difference between 24 and 96 h of culture (Fig. 6f). The red trend line (median ± median absolute deviation in 5 µm radially concentric layers) indicates a random isotropic orientation after 24 h of culture (≈ 45° and large median absolute deviation) while the trend line for MCTSs cultured for 96 h shows a higher degree of orientation that depends on the nucleus-perimeter distance (example shown in Supplementary Fig. 8b). The outermost layers, 0–35 µm, showed a significant difference between MCTSs cultured for 24 and 96 h.

Using the same 3D ellipsoid defined above, the nuclear aspect ratio was studied by the ratio between major axis (p_major_) and minor axis (p_minor_) lengths (Fig. 6g). The scatter plots show the major/minor axis ratio distribution as a function of nucleus-MCTS perimeter distance after 24 and 96 h (Fig. 6h). The red trend line (mean ± standard deviation in 5 µm radially concentric layers) indicates an even ratio around 2 for MCTSs cultured for 24 h while the nuclei in MCTSs cultured for 96 h has a slight ratio increase in the outer layers. There is a statistically relevant increase in major/minor axis ratio in the outer layers between 24 and 96 h of MCTS culture (Fig. 6i). The ratio between the major and intermediate axes lengths (Supplementary Fig. 9a) was found to be around 1.5 over the whole nucleus-MCTS perimeter distance covered and there were no statistically significant differences between the concentric layers in MCTSs cultured for 24 and 96 h (Supplementary Fig. 9b).

Since the resolution in the z-direction is not as good compared to the x–y optical section in confocal microscopy, possible imaging artefacts in the z-direction was studied by measuring the major, intermediate and minor axis lengths as a function of the z alignment angle. This angle was defined as the angle between the z-axis and the plane spanned by the major and intermediate principal axes having the same direction as the major and intermediate eigenvectors described above (Supplementary Fig. 10). A linear fit was used to calculate that there was a 2 µm major and minor axis length difference between a nucleus with 0° z alignment angle and a nucleus oriented 90° relative the z-axis. Overall, the principle axis lengths were 16.3 ± 2.3 µm, 11.8 ± 1.4 µm and 8.2 ± 1.2 µm for the major, intermediate and minor axes respectively (mean ± standard deviation, n = 6083).

## Discussion

In this study we focused on connecting nuclear segmentation data to cellular organization and the cell cycle phase using volumetric parameters and DNA-content. The nuclear segmentation and analysis were performed on z-stacks from cleared and DAPI-stained MCTSs imaged by confocal microscopy. The accuracy of the nuclear segmentation was confirmed by visual inspection, manual correction of non-segmented nuclei clusters and volume gating to discard out of bound objects. Analysis of the DAPI stained and FUCCI transfected MCTSs supports that the segmentation approach was accurate since the DNA-content correlates well with the cell cycle phase. As an example, the 1291 cells defined as G1 cells by FUCCI fluorescence were well collected into a diploid, 2N, distribution where only a few nuclei were found in the distribution tail towards 4N which could be accounted for by slight segmentation errors or additional copy numbers of DNA.

Polyploidy, more than the usual number of chromosomes (4N, 8N, 16N etc.), and aneuploidy, where the number of chromosomes are uneven, could be an issue when using DNA-content analysis to distinguish the cell cycle phase in cancer cell lines^45^. The polyploid cells could include two nuclei, which would be detected as two individual cells in our study since we do not stain the cell membrane, or a large single nucleus. The large single polyploid nuclei was weeded out by the volume gating and no polyploid cells (> 4N) were included in the DNA-content based cell cycle classification. Existence of aneuploid cells might lead to broadening of the DNA-content distribution but the combined FUCCI and DNA-content data used here indicated that it is possible to use DNA-content to classify the cell cycle in A498 cells. The polyploid fraction of A498 cells is unknown and the polyploid sensitivity in cell cycle classification might be cell line dependent.

While not in focus in this investigation, the polyploid population discarded in the post-segmentation analysis could be of interest for other types of studies. Aneuploid and polyploid cancer cells are known drivers of cancer therapy resistance and tumor repopulation after completed therapy^46,47^. Our DNA-content characterization method could easily be adjusted to focus on the presence and spatial organization of polyploid cells in MCTSs for future drug discovery and tumor therapy resistance investigations.

The use of a support vector machine (SVM) to improve the S/G2 classification precision compared to a regular threshold in the DNA-content histogram was successful. While the true positive rate dropped from 79.8 to 70.3%, it was balanced by a decreased false positive rate (from 6.3 to 1.4%) giving an overall improved precision of 86.7% compared to 62.6%, justifying the SVM classification. The DNA-content threshold for S/G2 classification should not be susceptible to cell line changes as long as the nuclear segmentation is accurate while the SVM model could be more sensitive to cell line dependent changes in nuclear morphology since volumetric data was used in the training. However, using volumetric data normalized to the neighboring cells in the SVM training to avoid spatial dependence in the classification model could also reduce some of the cell line sensitivity in our approach.

The main S/G2 classification error source, using both the DNA-content threshold and the SVM, stems from the early S phase cells (identified by FUCCI and 2N DNA content) which were similar to G1 cells in volumetric parameters. No nuclear texture parameters were used to quantify the spatial DNA distribution within the nuclei in this study but could in future work be included to investigate improved classification schemes.

The spatial distribution of S/G2 phase cells within the A498 MCTSs (Fig. 5) showed that the majority of cells were found in the outer layer but another picture arises when the number of S/G2 cells was normalized to the total number of cells within each layer. The fraction of S/G2 phase cells were found to be evenly distributed as a function of nucleus-MCTS perimeter distance with the exception for the inner core. The drop in proliferating cell fraction in the core is due to the small measurement volume which includes few cells. The even S/G2 phase cell fraction distribution is expected since the diameters of the MCTSs used in this study were between 120 and 150 µm (Supplementary Fig. 6) while the MCTS diameter should be around 500 µm to induce a necrotic core surrounded by a quiescent and proliferating layer^48^. The relatively even fraction distribution of S/G2 positive cells over the perimeter distance covered in this study has also been observed in Capan-2 MCTSs with FUCCI^11^.

The nuclear volume, aspect ratio and alignment angle as a function of nucleus-perimeter distance (Fig. 6) shows how volumetric parameters can be used to study MCTS organization. While a small nucleus volume decrease can be seen between 24 and 96 h, there was a distinct trend of reduced nucleus volume as function of distance from the MCTS perimeter. A large difference in alignment angle distribution between the two timepoints was also observed. A study measuring the deformation of MCTS embedded hydrogel beads with known mechanical properties showed that the mechanical stress increased towards the MCTS center which could explain the decreasing nucleus volume as function of distance to the MCTS surface shown in our investigation^49^. In the same study the authors also used phalloidin staining and found that the cell orientation depends on the position within the MCTS equatorial plane and correlates with the stress distribution. We show that we can use the nuclear alignment angle to accurately quantify the same phenomena in 3D and that the nuclear and cell organization depends on time in culture assuming that the cell body and nucleus deformation and orientation are equivalent. The difference in nuclear alignment angle between MCTSs cultured for 24 and 96 h could thus be related to the stress gradient developing over time. This is also supported by the decreased nucleus volume as a function of distance from the MCTS perimeter, as well as the increased ratio between major and minor axis lengths in MCTSs cultured for 96 h. On the other hand, the ratio between major and intermediate axis was unchanged between 24 and 96 h. Due to the nuclear alignment in MCTSs cultured for 96 h, the minor axis of the nuclei lies parallel to the radius and the stress gradient which decreases the nucleus thickness in the radial direction. The drop in the anisotropic nuclear alignment closer to the geometric MCTS center after 96 h could be explained by the stress gradient direction being less distinct because of the MCTS symmetry. Indirectly measuring the stress distribution and interstitial fluid pressure through the nucleus alignment angle and nucleus volume distribution could be a good control, or even target, in drug development studies. A high interstitial fluid pressure in tumors compared to healthy tissue is known to hamper therapy distribution and therefore treatment response^50^. It might even be possible to use nuclei within MCTSs as pressure sensors instead of hydrogel beads to extend our methodology from qualitative to quantitative pressure and stress measurements. While extracting the deformation of fluorescently stained hydrogel beads embedded into the MCTSs might be a more accurate tool to measure the stress gradient, using the nuclear segmentation data would add mechanical phenotyping within MCTSs without introducing foreign objects or extra fluorophores.

We have in this study applied our imaging, segmentation and analysis methods to MCTSs formed from cell lines and the methods outlined herein should work for MCTSs cultured form other cell lines as well. However, an attractive application for high content screening is personalized medicine investigations using MCTSs formed from patient samples. Patient tumor samples are complex and contains many different cell types. Future nuclear segmentation strategies should focus on handling nuclei of vastly different sizes. Also, aneuploidy and polyploidy are expected to vary more in tumor samples which might make the cell cycle analysis more complex. In future developments of the methods presented herein one could use the stromal cells in the tumor sample as a DNA index base line to evaluate the DNA index of the tumor cells^51^. By taking advantage of the stromal cells it might be possible to characterize the cell cycle or at least the DNA content in combination with the volumetric parameter data.

## Conclusion

We have shown that nuclear segmentation in MCTSs can be used to classify the cell cycle phase using volumetric and DNA-content data. Furthermore, the nuclear segmentation can also be used to study cellular organization in MCTSs. Using a single fluorophore, DAPI, there is a lot of biologically relevant information, such as cell cycle stage, nuclear volumetric parameters and cellular organization within the MCTSs, that can be acquired from standard counter-staining protocols while leaving the rest of the fluorescence spectrum free for other probes. The methodology presented herein is high-content and could be used with any accurate nuclear segmentation strategy and stochiometric DNA staining. We believe that the methodology outlined in this paper will be valuable in basic tumor biology research and cancer therapy applications.

## Supplementary Information

Supplementary Figures.

## Data Availability

The MATLAB code is available at https://github.com/Karlolo90/NuclearAnalysis.
