# Supplementary material for: Single cell organization and cell cycle characterization of DNA stained multicellular tumor spheroids
Source: Sci Rep. 2021 Aug 23;11:17076. doi: 10.1038/s41598-021-96288-6 (PMC8382712; doi:10.1038/s41598-021-96288-6)
Supplement: Supplementary file 1 — Supplementary Figures. [file 41598_2021_96288_MOESM1_ESM.pdf]

Supplementary Information: Single cell organization and cell cycle characterization of DNA stained multicellular tumor spheroids

Karl Olofsson[1], Valentina Carannante[2], Björn Önfelt[1,2] and Martin Wiklund[1]
[1]Dept. Of Applied Physics, Science for Life Laboratory, KTH Royal Institute of Technology, SWEDEN
[2]Dept. of Microbiology, Tumor and Cell Biology, Karolinska Institute, SWEDEN

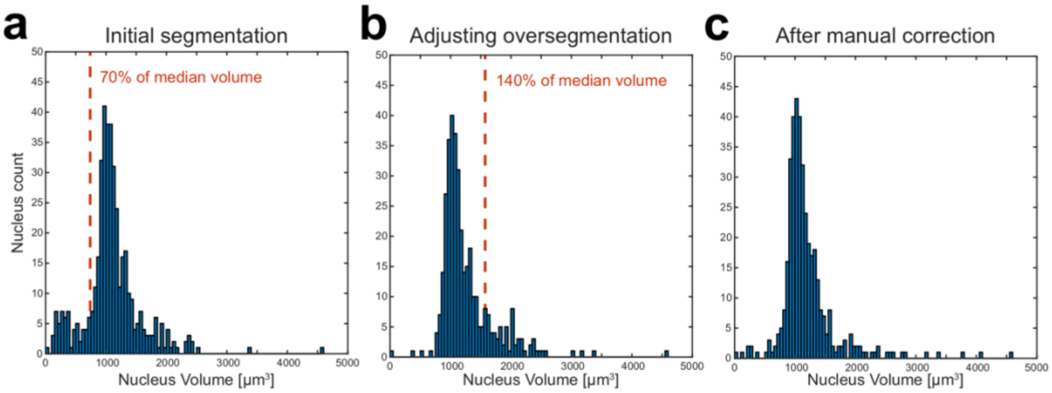

Supplementary Figure 1: Automatic and manual seed correction improves segmentation. Histograms show the nucleus volume distribution after initial segmentation **(a)**, over segmentation adjustment **(b)** and manual correction **(c)** for an individual multicellular tumor spheroid (MCTS). The dashed red line indicates the nucleus volume cut-off for over segmentation adjustment (< 70% of median volume **(a)**) and manual inspection and correction (> 140% of median volume **(b)**).

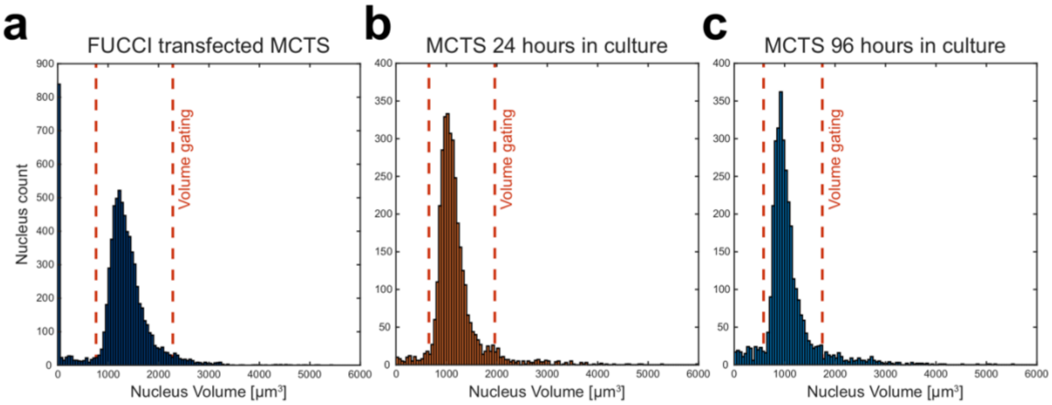

Supplementary Figure 2: Volume gating excludes small debris and non-segmented nuclei. Nucleus volume distributions show all the segmented objects in FUCCI transfected MCTS **(a)** and MCTS cultured for 24 hours **(b)** and 96 hours **(c)**. The dashed red lines indicate the volume gating (60% to 180% of the median nucleus volume) to exclude small debris and possible non-segmented nuclei clusters from post-segmentation analysis.

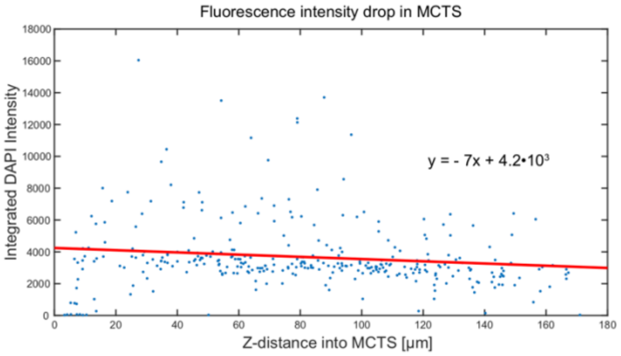

Supplementary Figure 3: Linear correction of fluorescence intensity drop for more accurate DNA-content measurements. The integrated DAPI intensity and imaging depth (z-distance) of each nucleus (blue dots) was used to linearly correct the DAPI intensity (red line) in each MCTS.

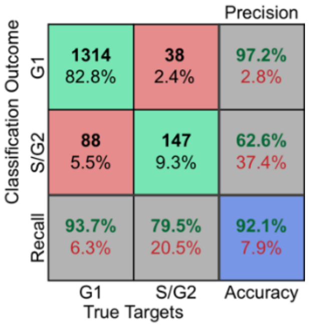

Supplementary Figure 4: Classification accuracy of integrated DAPI thresholding. The confusion matrix for the integrated DAPI threshold shows correctly (green boxes) and incorrectly (red boxes) classified G1 and S/G2 cells with the recall, precision (grey boxes) and overall accuracy (blue box).

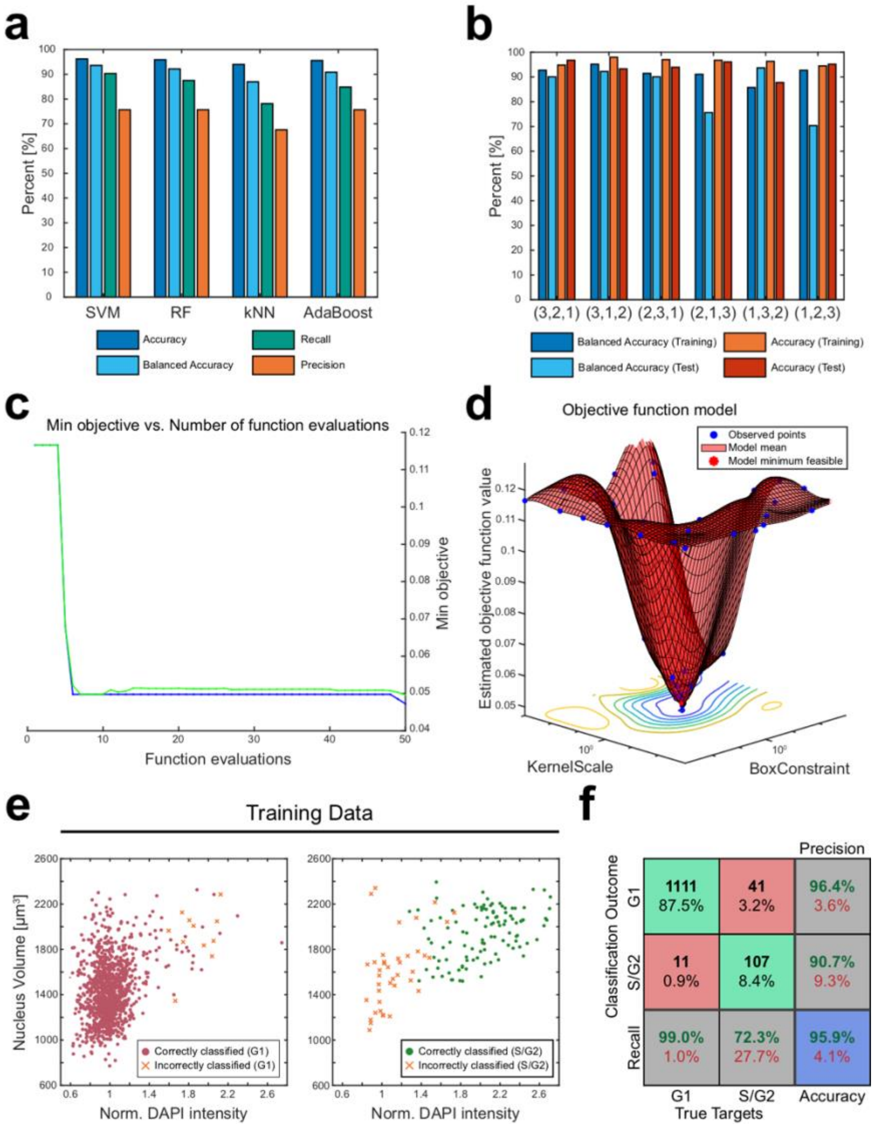

Supplementary Figure 5: Support vector machine (SVM) benchmarking, robustness test, hyperparameter optimization and training enable SVM S/G2 cell classification. The balanced accuracy, overall accuracy, precision and recall of the SVM model was compared to a random forest, k-nearest neighbor and adaBoost with decision trees algorithms **(a)**. The robustness in terms of balanced accuracy and overall accuracy for the SVM model was tested by splitting the data based on microplate (n_{plate1} = 222, n_{plate2} = 764 and n_{plate3} = 416) and optimizing, training and testing on all permutations of the data (i = optimization, j = training, k = testing) **(b)**. Using 80% of the available FUCCI labeled data, a Bayesian hyperparameter optimization of the SVM was performed over 30 iterations (**c**) yielding a model of optimal kernel scale and box constraint **(d)**. Scatterplots of integrated DAPI intensity per nucleus against volume show correctly classified G1 (red) and S/G2 (green) cells as dots and misclassified cells as orange crosses for the training data **(e)**. Confusion matrix describing the SVM classification performance shows the correctly (green) and incorrectly (red) classified cells, recall (true positive and false negative rate), precision (positive predictive value and false discovery rate) and overall accuracy (blue) after SVM training **(f)**.

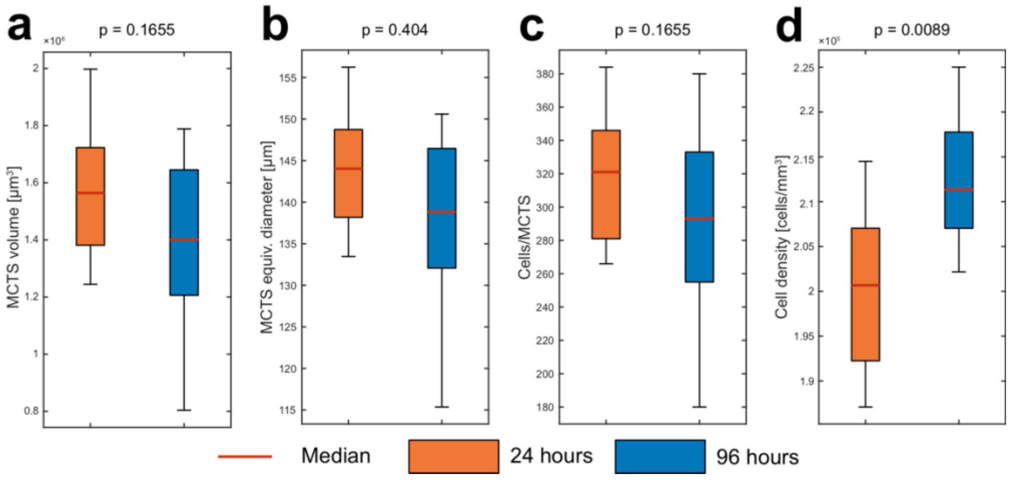

Supplementary Figure 6: A498 MCTS characteristics after 24 and 96 hours. A498 MCTSs fixed after 24 hours (orange bars, n = 10) and 96 hours (blue bars, n = 10) were characterized based on their volume **(a)**, diameter of a sphere with equal volume as the MCTS **(b)**, number of cells per MCTS **(c)** and cells per mm^3 **(d)**. Boxes show the 25th and 75th percentiles with median marked with red lines. Whiskers shows the furthest observation within 1.5 times the interquartile length away from the box edge. Statistical significance test by Mann-Whitney U-test and p-values are shown above the boxplots.

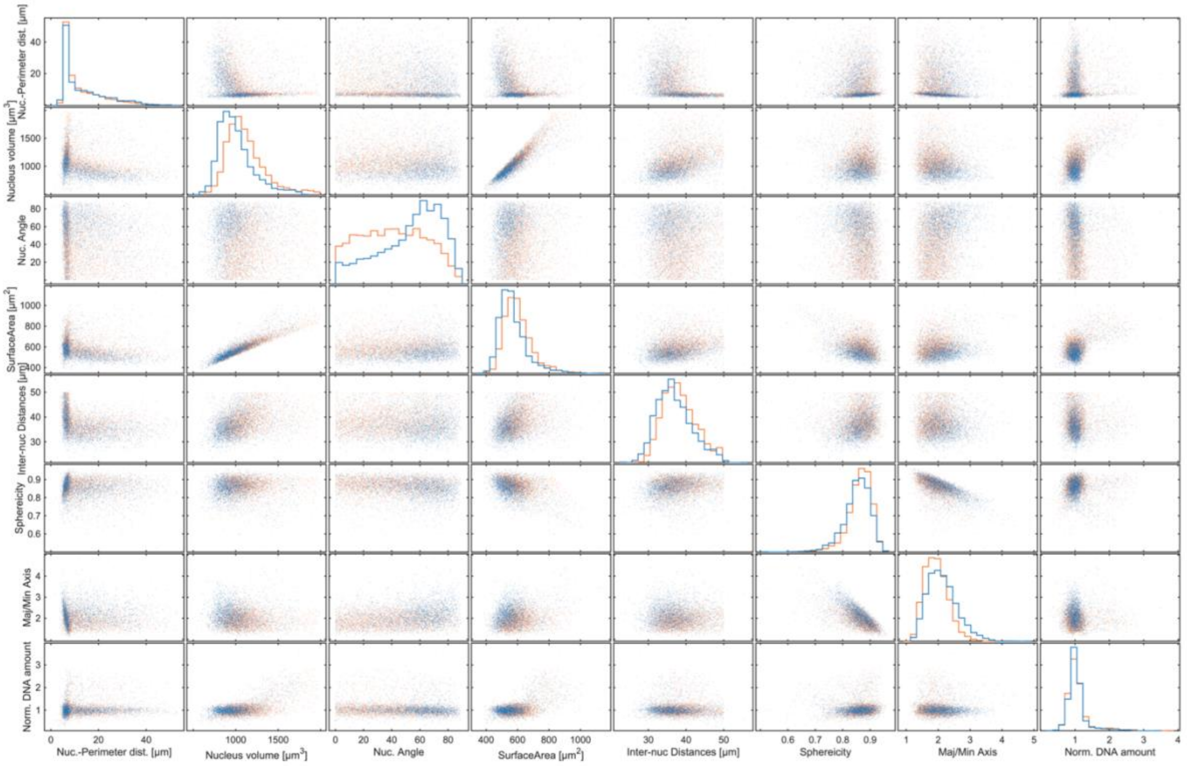

Supplementary Figure 7: Volumetric parameters can be used to investigate structural organization within MCTSs. A scatter plot matrix, with histograms along the diagonal, of volumetric parameters measured on nuclei segmented from MCSTs culture for 24 hours (orange dots) and 96 hours (blue dots).

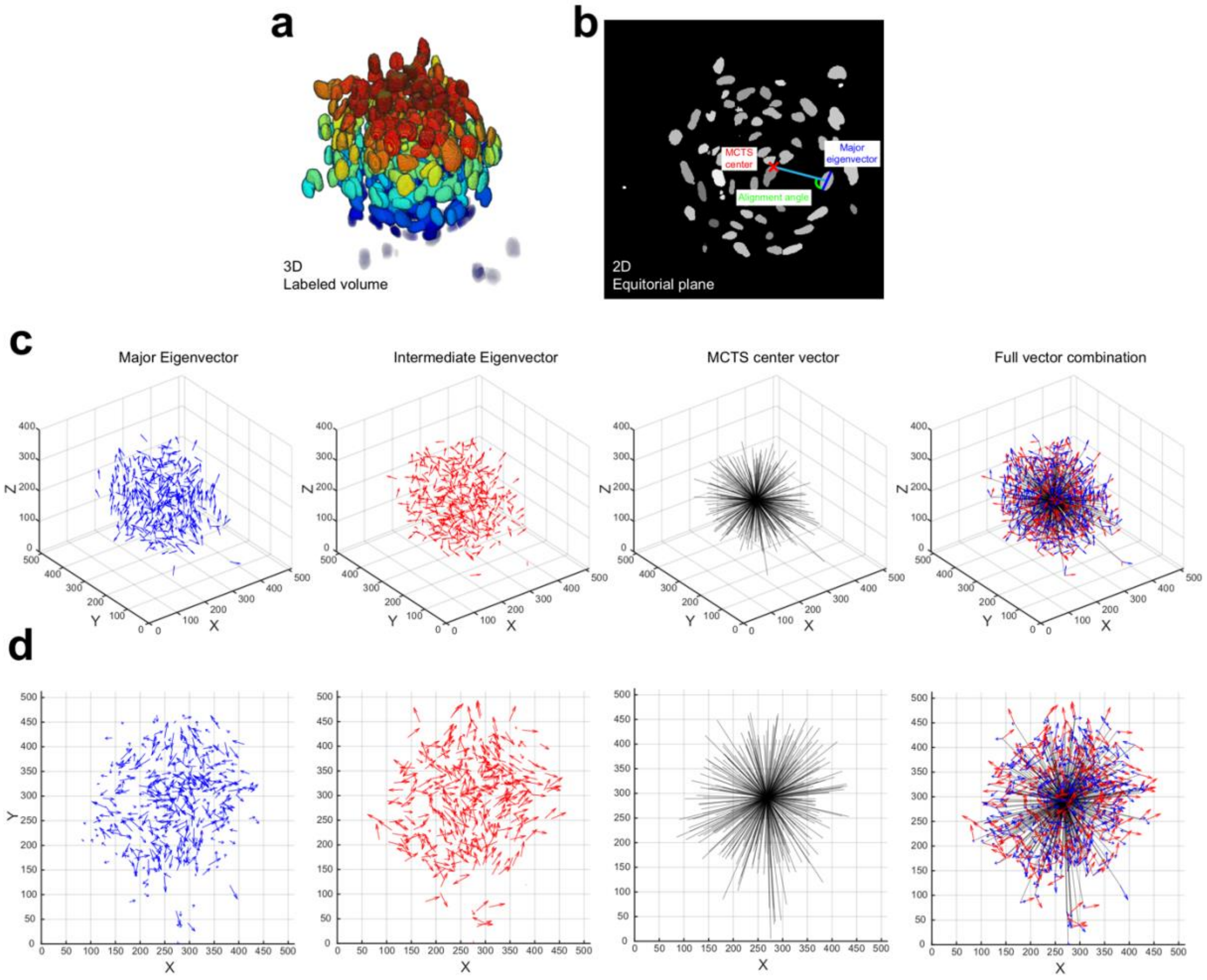

Supplementary Figure 8: Nucleus alignment angle against the MCTS center point can be used to study nuclear orientation. Each nucleus within the segmented and labeled MCTSs **(a)** was characterized in terms of nucleus alignment angle (schematically shown in 2D **(b)**). The nucleus alignment angle was measured as the angle between the plane spanned by the major and intermediate eigenvectors and the vector between the MCTS center and nucleus center (3D in **(c)** and superimposed in the XY plane **(d)**).

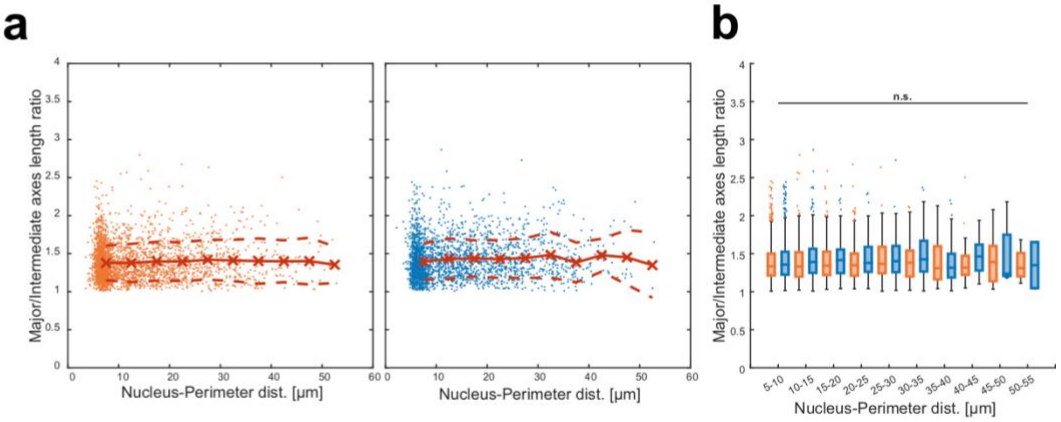

Supplementary Figure 9: The ratio between major and intermediate principal axis lengths does not depend on nucleus position. Scatterplots shows the nucleus-MCTS perimeter distance against the ratio between the major and intermediate principal axis lengths in MCTSs cultured for 24 hours (orange dots) and 95 hours (blue dots) **(a)**. The trend line (solid red) and standard deviation (dashed red) was calculated as mean and standard deviation in 5 µm thick concentric layers. Box plot charts summarizes the data side by side for easier comparison **(b)**. Significance was tested with two-way ANOVA followed by Tukey's post-hoc multiple comparison (**: $p < 0.001$).

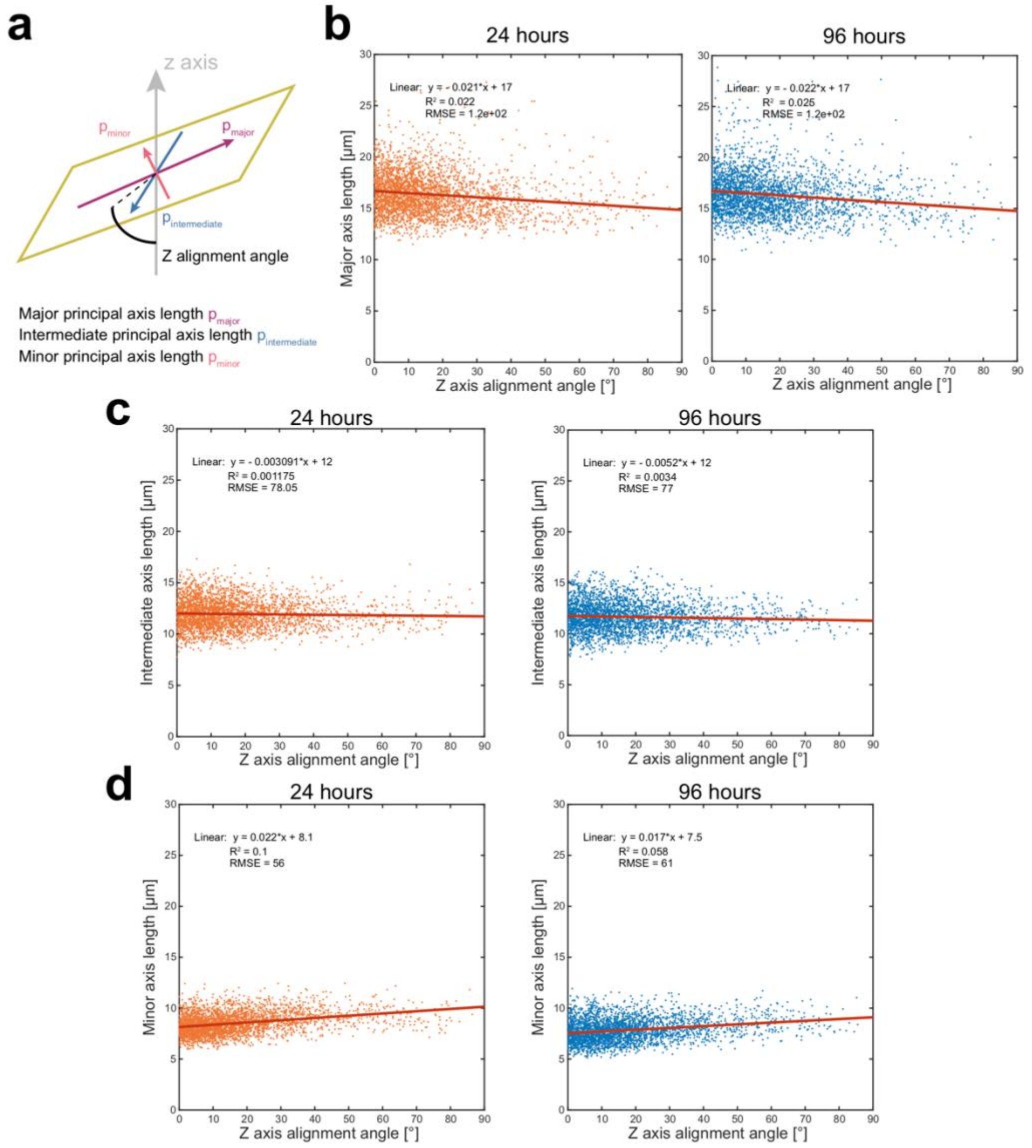

Supplementary Figure 10: Imaging artefacts in the z-axis direction. The imaging artefacts due to different resolution along the z-axis was studied by investigating the major, intermediate and minor principal axis lengths as a function of nucleus orientation relative to the z-axis **(a)**. The z alignment angle was determined by measuring the angle between the z-axis and the plane spanned by the major and intermediate principle axes. All axis lengths in nuclei from MCTSs cultured for 24 hours (orange dots) and 96 hours (blue dots) was measured and plotted against the z alignment angle **(b-d)**. The red line in the scatterplots show the linear fit with the function and goodness of fit specified in the upper left corner.
